# HPB News

**DOI:** 10.1155/1992/37048

**Published:** 1992


					HPB Surgery, 1992, Vol. 5, p. 215
Reprints available directly from the publisher
Photocopying permitted by license only
1992 Harwood Academic Publishers GmbH
Printed in the United Kingdom
HPB NEWS
INTERNATIONAL HEPATO-BILIARY-PANCREATIC ASSOCIATION
FRANK GLENN TRAVELLING FELLOWSHIP 1992
The International Hepato-Biliary-Pancreatic Association invites applications for
the above travelling Fellowship. A stipend of $5000 Dollars U.S. is available to
subsidize a young investigator in the field of hepato-biliary and pancreatic dis-
orders. The applicant must be sponsored by a member of the Association. The
stipend may be used to support a laboratory project and/or to subsidize travel
expenses which are deemed appropriate to the field of investigation. Applicants
must be under 40 years of age and should have an established record of at least one
or several papers concerning liver, biliary or pancreatic disease.
Applications should be made in writing and should include the following:
1. a copy of the applicant's curriculum vitae;
2. a summary of 1-2 pages of a research protocol describing how the stipend is to
be used;
3. a letter of nomination from a member of the IHBPA Association or your
Department Chairman.
Send the application to Haile T. Debas, M.D., University of California, San
Francisco, Department of Surgery, San Francisco, CA 94143-0104, USA. by 30
April 1992.
TEL: (415) 476-1236 FAX: (415) 476-1734.
215

				

